# Impacts of Educational Interventions with Support of Mobile App versus Booklet for Patients with Hypertension and Metabolic Syndrome: A Secondary Data Analysis

**DOI:** 10.3390/ijerph191912591

**Published:** 2022-10-02

**Authors:** Eliza Mi Ling Wong, Hon Lon Tam, Angela Yee Man Leung, Alice Siu Ping Cheung, Ka Ching Cheung, Doris Yin Ping Leung

**Affiliations:** 1 School of Nursing, Tung Wah College, Hong Kong SAR, China; 2 The Nethersole School of Nursing, The Chinese University of Hong Kong, Hong Kong SAR, China; 3 School of Nursing, The Hong Kong Polytechnic University, Hong Kong SAR, China

**Keywords:** metabolic syndrome, hypertension, educational intervention, mhealth, body weight, exercise, self-efficacy

## Abstract

Background: Hypertension comorbid with metabolic syndrome could increase the development of adverse cardiovascular events. Educational interventions were effective to improve outcomes in patients. Methods: This was a secondary data analysis of participants with hypertension. The original randomized controlled trial aimed to examine the effect of app and booklet versus control among individuals diagnosed with metabolic syndrome living in the community. A 30-min health education was provided to each participant. In addition to the education, the app group received a mobile app while the booklet group received a booklet. Data were collected at baseline, week 4, week 12, and week 24. Intention-to-treat principle was followed, and generalized estimating equations was employed for data analysis. Results: A total of 118 participants with hypertension and metabolic syndrome were extracted from the three-arm trial data. The sample size was 36, 42, and 40 in the app group, booklet group, and control group, respectively. Compared to the control group, the app group showed a significant reduction on body weight and waist circumference at week 24, while the total exercise and self-efficacy for exercise were increased at week 12 and week 24 but no significant findings were observed in the booklet group. Conclusions: The educational intervention supported with app was superior to the booklet support on the outcomes of body weight, waist circumference, total exercise, and self-efficacy for exercise among patients with hypertension and metabolic syndrome in the community.

## 1. Introduction

A person with metabolic syndrome (MetS) should have a cluster of cardiometabolic risk factors such as central obesity (defined as waist circumference with ethnicity-specific values) plus two of the follow factors or be receiving related treatment, such as dyslipidemia, insulin resistance, and hypertension [1]. Although the diagnostic criteria of MetS are different between professional institutions, it was estimated that over 1 billion people were having MetS worldwide [2,3]. The prevalence of MetS has been increasing, with prevalence of over 30% in the western population and about 22% in the Chinese population [2,4]. If MetS is not well controlled, it leads to coronary heart disease and stroke [5,6]. Regarding the diagnostic criteria of MetS, hypertension is raised concern. Hypertension is first among the risk factors to cause health burden worldwide that led to various adverse cardiovascular events, such as heart diseases and stroke, and attributed 10.8 million global deaths in 2019 [7,8]. The American Heart Association indicates that MetS amplifies the chance of developing adverse cardiovascular events [9].

Lifestyle modifications in terms of healthy diet and regular exercise are recognized important in the management of hypertension and MetS [1,3,10,11,12]. Educational interventions, such as health education and booklet, could effectively improve the adherence to lifestyle modifications [13,14,15]. With the popularity of smartphone ownership, mobile apps have been developed to provide support to health education [16,17]. These support program have proven effective to support for continuous care for patients with MetS or cardiac risks [17,18,19,20,21]. However, these reviews and studies focused on either hypertension or MetS. Patients with hypertension and MetS are concerned since they have higher chance of adverse cardiovascular events than other MetS patients without hypertension [5]. Up to our knowledge, there is no study examined the effect of app, booklet versus control on patients with hypertension and MetS. We intend to carry out a secondary data analysis to examine the outcomes for these group of patients.

### Aims

The secondary data analysis aimed to examine the effect of an educational intervention using mobile app and booklet versus control group, and mobile app versus booklet on outcomes of body weight, total exercise, cardiometabolic profile (waist circumference, blood pressure, cholesterols, triacylglycerols, and fasting blood glucose), perceived stress, and exercise self-efficacy for patients with hypertension and MetS in the community.

## 2. Materials and Methods

This was a sub-group analysis of a three-arm randomized controlled trial (RCT). The aim of the original three-arm RCT was to compare the effect a lifestyle intervention using mobile app versus booklet for MetS patients living in the community. The trial has been registered in ClinicalTrials.gov with ID: NCT03778788, and the study protocol has been published elsewhere [22]. Eligible participants were randomly assigned to the app group, booklet group, and the control group in a ratio of 1:1:1 with outcome assessments at baseline (T1), week 4 (T2), week 12 (T3), and week 24 (T4).

### 2.1. Participants and Inclusion and Exclusion Criteria

Data were collected from August 2019 to December 2021 in Hong Kong. Participants were recruited at two community centers located near the participating university using convenience sampling. 368 people were screened, 264 of them had MetS and agreed to participate the study. 118 participants with hypertension and MetS were selected for this secondary data analysis. The sample size was 36, 42, and 40 in the app group, booklet group, and control group, respectively.

### 2.2. Intervention Material

The interventions for each group followed the published study protocol [22]. Self-efficacy theory and health belief model are the theoretical framework used to guide the development of interventions for app group and booklet group. Participants in the same group were called back in a day to receive a standard 30-min group health education related to healthy lifestyle delivered by a trained nurse. Brisk walking exercise was suggested in the education. Different intervention days were assigned to specific groups to minimize subject contamination. 

App group. A research assistant (RA) assisted participants to install a MetS mobile app and explain the use of app after the health education. Then, the participants could read the same knowledge content as the booklet in the app. A membership area provided individual support for self-health monitoring, goal setting for their exercise plan and exercise record. 15 daily messages were delivered the participants to enhance their interest in the app intervention. Congratulatory remarks were provided if the participants completed their exercise goal, and an exercise reminder was sent if they did not enter the exercise record for two weeks. 

Booklet group. The participants received a healthy lifestyle booklet and read it at home. The booklet covered the same content of the facts about MetS and advice on diet, exercise, medication, lifestyle, and stress management. 

Control group. A placebo leaflet containing information on obesity and healthy lifestyle was delivered to the participants. For ethical reasons, the participants received the healthy lifestyle booklet after completion of the study.

### 2.3. Data Collection

The eligible participants signed the written consent before baseline assessment. Outcomes were assessed again at T2, T3, and T4. Two RAs collected data, one for baseline and one for the other three assessments after randomization. Both were blinded to the study allocation and received training for data collection, and the participants in each group were arranged on different date for follow-up assessments to avoid contamination.

### 2.4. Outcvome Variables

Details of primary outcome and secondary outcomes were described in the published study protocol [22]. In brief, body weight was the primary outcome that was measured by the same electronic weight scale. The secondary outcomes were described as follows.

Total exercise was measured by a modified version of the Godin-Shephard Leisure-Time Physical Activity Questionnaire (GSLTPAQ), which assessed the amount and difficulty of the exercises performed [23]. Cardiometabolic profile in terms of waist circumference, blood pressure, total cholesterol, high density lipoprotein (HDL) cholesterol, low density lipoprotein (LDL) cholesterol, triacylglycerols, and fasting blood glucose were measured by validated monitor. Three-min step test was used to assess the participant’s cardiovascular functional endurance. The perceived stress scale (PSS-10), a 4-point Likert scale with 10 items, used to assess the participant’s stress [24]. The total scores range from 0 to 40 that a higher score indicates a higher level of stress. Exercise self-efficacy was measured by a validated scale, self-efficacy for exercise scale (SEE) [25]. The scale consists of 9 items with total scores range from 0 to 90. A high score indicates a high level of self-efficacy to do exercise. All of the primary outcome and secondary outcomes were measured at T1, T2, T3, and T4, except cardiometabolic profile and three-min step test were measured at T1 and T4 only.

### 2.5. Data Analysis

Data were analyzed using SPSS version 26. Baseline characteristics and outcome variables were compared between the three groups using chi-square tests for categorical variables or ANOVA for continuous variables. By following the intention-to-treat principle, generalized estimating equation (GEE) was adopted to compare the mean changes of the app group and booklet group to the control group in the continuous variables. All statistical tests were two-sided and a *p*-value < 0.05 was considered as statistical significance. Between-group effect size at T4, measured by Cohen’s d, on each outcome variable was determined according to the following: small = 0.2; moderate = 0.5; and large = 0.8 [26].

## 3. Results

### 3.1. Baseline Characteristics

Table 1 shows the sociodemographic and clinical characteristics of the participants among the three groups. The mean age was 65.72 ± 9.25 years. Most of the participants were female (*n* = 83, 70.33%), educated at secondary school and higher (*n* = 91, 77.12%), married (*n* = 72, 61.02%), and retried (*n* = 66, 55.93%). The mean value of outcome variables at baseline was: body weight = 69.43 ± 14.43 kg, GSLTPAQ = 14.43 ± 16.25, waist circumference = 95.68 ± 9.52 cm, systolic blood pressure (SBP) = 135.44 ± 14.44 mmHg, DBP = 82.81 ± 9.64 mmHg, total cholesterol = 4.69 ± 1.12 mmol/L, HDL cholesterol = 1.32 ± 0.32 mmol/L, LDL cholesterol = 2.53 ± 0.97 mmol/L, triacylglycerols = 1.73 ± 0.77 mmol/L, fasting blood sugar = 6.20 ± 1.50 mmol/L, 3-min step test = 4.19 ± 1.40, PSS-10 = 15.07 ± 5.43, SEE = 4.63 ± 1.87. Regarding the outcome variables at baseline, no significant difference was noted, except DBP. The mean DBP in the control group (79.05 ± 10.63 mmHg) was statistically lower than the app group (83.42 ± 10.17 mmHg) and booklet group (85.86 ± 6.77 mmHg). 

### 3.2. Efficacy of Different Interventions on Outcome Variables at Follow-Ups

**Figure 1:**
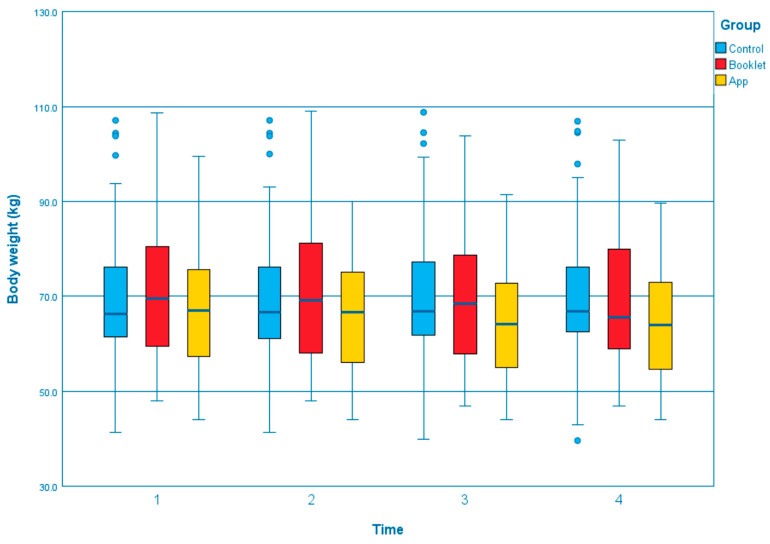
Boxplot of the change of body weight at different time point among the groups.

**Figure 2:**
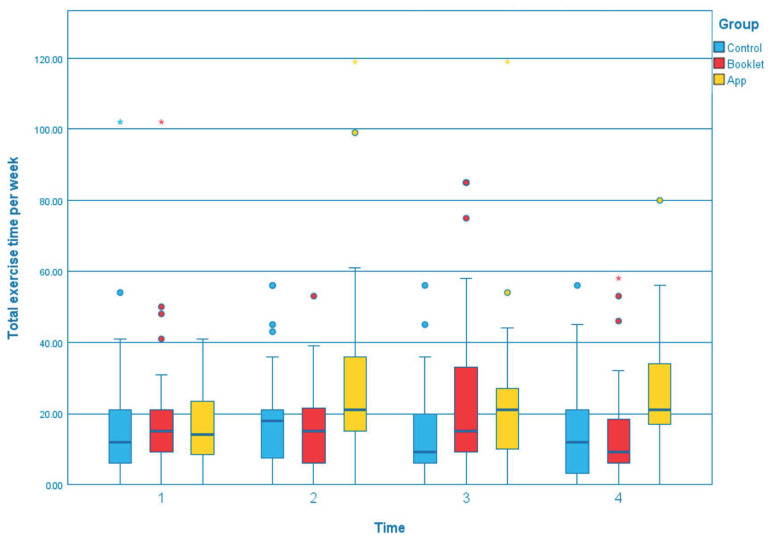
Boxplot of the change of total exercise time per week at different time point among the groups.

**Figure 3:**
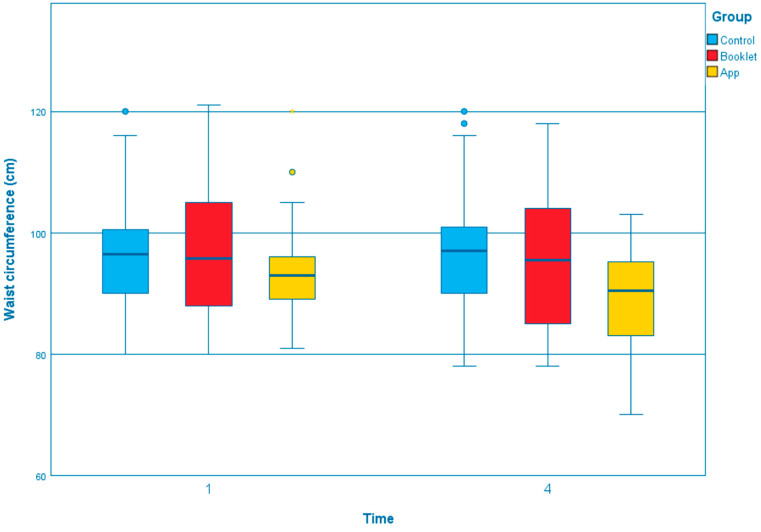
Boxplot of the change of waist circumference at different time point among the groups.

**Figure 4:**
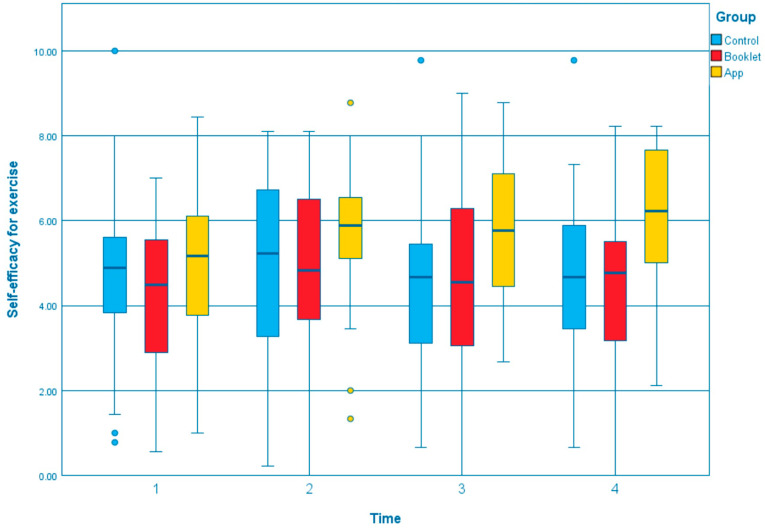
Boxplot of the change of self-efficacy for exercise at different time point among the groups.

The GEE results of outcomes across T1, T2, T3, and T4 between three groups were summarized in Supplementary materials, Tables S1 and S2. Compared to the control group, the app group showed a significant effect on several outcomes that the body weight was reduced significantly (Figure 1; T3, β = −0.913, *p* = 0.040; T4, β = −1.254, *p* = 0.007). The changes in total exercise time per week, measured by GSLTPAQ, indicated that the app group could significantly increase in exercise time at all of the time points than the control group (Figure 2; T2, β = 11.647, *p* = 0.032; T3, β = 10.141, *p* = 0.041; T4, β = 11.784, *p* = 0.013). Regarding the waist circumference, both the booklet group and app group showed greater reductions than the control group (Figure 3). However, only the reduction in the app group was statistically and significantly different from the control group at T4 (β = −3.842, *p* < 0.001). Self-efficacy for exercise in the app group was improved significantly than the control group at T3 (β = 1.043, *p* = 0.037) and T4 (β = 1.170, *p* = 0.031), Figure 4. Regarding the comparison between the app group and booklet group (Table S2), results showed that the use of app could significantly increase total exercise time per week (T4, β =14.709, *p* = 0.001) and reduce waist circumference (T4, β = −2.688, *p* = 0.015).

Table 2 shows the effect size estimations of the app and booklet on the outcome variables at T4. As compared to the control group, the app group has better performance than the booklet group in most outcomes. The app group had a moderate-to-large effect to reduce body weight (Cohen’s d = −0.673), a large effect to reduce waist circumference (Cohen’s d = −1.197), and a small-to-moderate effect to improve total exercise time per week (GSLTPAQ, Cohen’s d = 0.495). Regarding the reduction of total cholesterol, compared to the control group, the booklet group (Cohen’s d = −0.421) showed a better effect than the app group (Cohen’s d = −0.349). On the other hand, the app group had no effect with regards to reducing stress (Cohen’s d = 0.038), but the booklet group had a small-to-moderate effect in terms of reducing stress (Cohen’s d = −0.344).

## 4. Discussion

To the best of our knowledge, this is the first analysis to evaluate the effect of theory-guided app and booklet versus control group among patients with hypertension and MetS in community. In the sub-group analysis of the three-arm RCT, the theory-guide MetS app could significantly increase the participant’s total exercise and self-efficacy to do exercise. As a result, their body weight and waist circumference were reduced significantly after 24 weeks. The MetS app had moderate-to-large effect on the reduction of body weight and waist circumference and small-to-moderate effect to improve total exercise time per week. Two systematic reviews reported that an e-health intervention with features of self-health monitoring, goal setting, and feedback would promote a behavior to become habit, such as doing more exercise and for a longer duration [21,27]. The membership area of the MetS app provides the forementioned features which may have further motivated the participant’s self-efficacy to do more exercise to reduce body weight and waist circumference.

Patients with hypertension and MetS did more exercises with the support of the MetS app. Two national studies found that patients with hypertension became physical inactive after the use of anti-hypertensive medication [28,29]. In addition to overweight and obesity, physical inactivity increases the likelihood of dyslipidemia, insulin resistance, and hypertension [30], which are the cardiometabolic risk factors of MetS [1]. By using the MetS app, patients with hypertension and MetS can be reversed from MetS to non-MetS as their body weight and waist circumference were reduced significantly. The change to non-MetS helps them to lower the chance of developing adverse cardiovascular events.

Except for waist circumference, no statistical significance was noted on other variables of cardiometabolic profile. In addition to the small number of participants in this secondary data analysis, the non-significant result might be attributable to the controlled values of the cardiometabolic profiles of the participants at baseline. The hypertension management guidelines indicated that the blood pressure for treated patients with hypertension should be controlled at SBP < 140 mmHg and DBP < 90 mmHg [10,11,12]. At baseline, the participants had already had a controlled blood pressure that might be difficult to detect significant change after the intervention. Regarding the blood analysis of cholesterols, the app might reduce the participant’s total cholesterol and LDL level and increase the HDL level. The non-significance results of cholesterols suggest an emphasis on healthy diet in the app in future. In turn, blood pressure and cholesterols are major predictors of cardiovascular risk [31]. The MetS app could potentially reduce the cardiovascular risk among patients with hypertension and MetS.

The GEE results showed that the participant’s stress level was decreased after the interventions. It was interesting that the reduction of stress in the booklet group was greater than that in the app group, compared to the control group. Furthermore, the booklet group showed a small-to-moderate effect to reduce stress. Although the content of stress management is included in both booklet and MetS app, the required digital competency in using the app might be another source of stressor [32]. In addition to one session to explain the use of app at the beginning, some follow-up calls or face-to-face sessions might be required to support the users proactively. On the other hand, the participants had already had a low level of stress at baseline. Future study can examine if the app and booklet are useful for patients at moderate or high level of stress.

### 4.1. Limitations

There are several limitations of this study. The sub-group analysis of a RCT and the groups for the current analysis were not generated by randomization and hence might have selection bias, although there was no statistical difference at baseline across groups. The small sample size was another limitation. Hence, only preliminary evidence is provided. Social desirability bias cannot not be compromised since several outcomes, including GSLTPAQ, PSS-10, and SEE, are self-reported. Step count device may be useful to provide objective measure of exercise. Female were dominant in this study, so the findings should be interpreted with caution. Lastly, this study is a secondary data analysis of a three-arm RCT and hence the conclusion of the results was preliminary. A full scale RCT for patients with hypertension and MetS is suggested for future research.

### 4.2. Implications

This study contributed to more evidence-based practice by examining the effect of a theory-guided intervention using a MetS app and a booklet versus a control group for patients with hypertension and MetS. The age of the participants indicated that the MetS app was suitable for older adults. The MetS app is important for MetS patients with obesity and hypertension, as these risks may be amenable due to our intervention. The educational intervention delivered by app could improve exercise self-efficacy, exercise amount leading to decreasing body weight, and waist circumference.

## 5. Conclusions

Patients with hypertension and MetS have a higher risk to develop adverse cardiovascular events yet amenable with life- style intervention. This study showed that the use of MetS app could significantly reduce their body weight and waist circumference and increase their exercise. The blood analysis of cholesterols was also improved. Considering the convenience, the app could be applied in the community care support to improve the patient outcomes.

## Figures and Tables

**Table 1:** Characteristics of participants and outcome variables at baseline.

Variables	App Group (*n* = 36)	Booklet Group (*n* = 42)	Control Group (*n* = 40)	*p*-Value
Age in years (SD)	66.94 (10.51)	64.07 (7.02)	66.35 (10.04)	0.344
Sex				0.156
Male	15	11	9	
Female	21	31	31	
Education level				0.09
Primary school or below	9	5	13	
Secondary school	16	29	20	
Tertiary education	11	8	7	
Marital Status				0.113
Married	25	26	21	
Not married	7	6	4	
Separated/Divorced/Widow	4	10	15	
Employment status				0.207
Full-time job	7	6	10	
Part-time job	3	6	1	
Housewife	3	10	6	
Retired/Others	23	20	23	
Financial status				0.118
Good	7	3	7	
Average	23	34	22	
Poor	6	5	11	
Residential status				0.106
Live alone	5	11	14	
Live with family	31	31	26	
Smoking status				0.607
Current smoker	0	0	1	
Quitted	3	6	4	
Never smoked	33	36	35	
Outcome variables (SD)				
Body weight	67.01 (12.76)	71.06 (14.88)	69.91 (15.41)	0.455
GSLTPAQ	17.18 (11.14)	18.07 (17.69)	16.97 (18.72)	0.949
Waist circumference	93.51 (8.30)	96.99 (10.44)	96.25 (9.41)	0.247
Systolic blood pressure	136.83 (15.07)	136.85 (14.51)	132.70 (13.76)	0.339
Diastolic blood pressure	83.42 (10.17)	85.86 (6.77)	79.05 (10.63)	0.004
Total cholesterol	4.68 (1.11)	4.91 (1.27)	4.49 (0.93)	0.233
HDL cholesterol	1.36 (0.33)	1.35 (0.34)	1.39 (0.31)	0.846
LDL cholesterol	2.55 (0.98)	2.70 (1.02)	2.32 (0.91)	0.213
Triacylglycerols	1.65 (0.77)	1.85 (0.82)	1.67 (0.72)	0.443
Fasting blood sugar	5.97 (0.70)	6.21 (1.43)	6.39 (2.02)	0.465
3-min step test	4.19 (1.39)	3.95 (1.28)	4.45 (1.53)	0.281
Perceived stress scale	13.58 (5.54)	15.66 (4.54)	15.77 (6.02)	0.143
Self-efficacy for exercise scale	4.95 (1.81)	4.24 (1.71)	4.76 (2.04)	0.21

GSLTPAQ = Godin-Shephard Leisure-Time Physical Activity Questionnaire, HDL = high density lipoprotein, LDL = low density lipoprotein.

**Table 2:** Comparing the effects of the app and booklet group at week 24.

Variables	Cohen’s d		
	App vs. Control	Booklet vs. Control	App vs. Booklet
Body weight	−0.673	−0.294	−0.360
Godin-Shephard Leisure-Time Physical Activity Questionnaire	0.495	−0.074	0.910
Waist circumference	−1.197	−0.488	−0.587
Systolic blood pressure	−0.005	0.009	−0.012
Diastolic blood pressure	−0.156	−0.296	0.111
Total cholesterol	−0.349	−0.421	0.038
High density lipoprotein (HDL) cholesterol	0.022	−0.241	0.239
Low density lipoprotein (LDL) cholesterol	−0.727	−0.248	−0.310
Triacylglycerols	0.301	0.090	0.160
Fasting blood sugar	0.177	0.151	0.056
3-min step test	0.015	0.027	−0.011
Perceived stress scale	0.038	−0.344	0.600
Self-efficacy for exercise scale	0.256	0.118	0.173

## Data Availability

Data are available upon request.

## Supplementary Materials

The following supporting information can be downloaded at: https://www.mdpi.com/article/10.3390/ijerph191912591/s1, Table S1: Results of generalized estimating equation analysis on outcome variables compared to the control group; Table S2: Results of generalized estimating equation analysis on outcome variables compared between the app group and booklet group.
