# Supplementary material for: Impacts of Educational Interventions with Support of Mobile App versus Booklet for Patients with Hypertension and Metabolic Syndrome: A Secondary Data Analysis
Source: Int J Environ Res Public Health. 2022 Oct 2;19(19):12591. doi: 10.3390/ijerph191912591 (PMC9565212; doi:10.3390/ijerph191912591)
Supplement: Supplementary file 1 [file ijerph-19-12591-s001.zip › ijerph-1903922-supplementary.pdf]

Table S1. Results of generalized estimating equation analysis on outcome variables compared to the control group.

Variables	Beta	Standard Error	95% Confidence interval		Wald Chi-square	df	p-value
			lower	upper			
Body weight							
Booklet group	1.152	3.307	-5.330	7.634	0.121	1	0.728
App group	-2.896	3.191	-9.152	3.360	0.823	1	0.364
T2	-0.060	0.110	-0.277	0.157	0.293	1	0.588
T3	-0.052	0.282	-0.605	0.501	0.033	1	0.855
T4	0.175	0.291	-0.396	0.747	0.362	1	0.547
Booklet group * T2	0.513	0.542	-0.550	1.577	0.895	1	0.344
Booklet group * T3	-0.190	0.585	-1.338	0.957	0.106	1	0.745
Booklet group * T4	-0.151	0.615	-1.358	1.055	0.061	1	0.806
App group * T2	-0.288	0.183	-0.647	0.070	2.485	1	0.115
App group * T3	-0.913	0.445	-1.785	-0.040	4.206	1	0.040
App group * T4	-1.254	0.465	-2.167	-0.341	7.242	1	0.007
GSLTPAQ							
Booklet group	1.096	3.978	-6.700	8.893	0.076	1	0.783
App group	0.206	3.449	-6.555	6.966	0.004	1	0.952
T2	1.075	3.271	-5.338	7.488	0.108	1	0.742
T3	-3.600	3.337	-10.141	2.942	1.163	1	0.281
T4	-2.172	3.462	-8.957	4.614	0.393	1	0.531
Booklet group * T2	-3.067	4.309	-11.514	5.381	0.506	1	0.477
Booklet group * T3	9.189	5.527	-1.645	20.023	2.763	1	0.096
Booklet group * T4	-1.132	4.249	-9.460	7.196	0.071	1	0.790
App group * T2	11.647	5.427	1.008	22.286	4.604	1	0.032
App group * T3	10.141	4.968	0.403	19.880	4.166	1	0.041
App group * T4	11.784	4.728	2.515	21.052	6.209	1	0.013
Waist circumference							
Booklet group	0.740	2.166	-3.507	4.987	0.117	1	0.733
App group	-2.736	2.005	-6.667	1.195	1.861	1	0.173
T4	0.630	0.522	-0.394	1.654	1.452	1	0.228
Booklet group * T4	-1.094	0.959	-2.974	0.786	1.301	1	0.254
App group * T4	-3.842	0.927	-5.659	-2.025	17.172	1	<0.001
Systolic blood pressure							
Booklet group	4.157	3.083	-1.886	10.201	1.818	1	0.178
App group	4.133	3.278	-2.293	10.559	1.589	1	0.207
T4	-0.521	2.174	-4.784	3.741	0.057	1	0.811
Booklet group * T4	0.119	3.427	-6.598	6.837	0.001	1	0.972
App group * T4	-0.060	2.949	-5.840	5.720	0.000	1	0.984
Diastolic blood pressure							
Booklet group	6.807	1.954	2.976	10.638	12.129	1	<0.001
App group	4.367	2.355	-0.250	8.983	3.437	1	0.064

T4	-0.786	1.140	-3.022	1.450	0.475	1	0.491
Booklet group * T4	-2.626	1.811	-6.176	0.925	2.101	1	0.147
App group * T4	-1.552	1.807	-5.094	1.990	0.737	1	0.391
Total cholesterol							
Booklet group	0.423	0.242	-0.052	0.879	3.052	1	0.081
App group	0.194	0.233	-0.264	0.652	0.692	1	0.406
T4	-0.030	0.042	-0.113	0.052	0.513	1	0.474
Booklet group * T4	-0.109	0.088	-0.284	0.065	1.517	1	0.218
App group * T4	-0.092	0.042	-0.342	0.158	0.524	1	0.469
HDL cholesterol							
Booklet group	-0.041	0.071	-0.180	0.098	0.338	1	0.561
App group	-0.028	0.072	-0.171	0.115	0.146	1	0.702
T4	-0.014	0.026	-0.066	0.038	0.286	1	0.593
Booklet group * T4	-0.039	0.0391	-0.116	0.038	1.001	1	0.317
App group * T4	0.004	0.037	-0.071	0.078	0.010	1	0.920
LDL cholesterol							
Booklet group	0.379	0.210	-0.035	0.792	3.227	1	0.072
App group	0.228	0.216	-0.196	0.651	1.107	1	0.293
T4	0.005	0.046	-0.086	0.095	0.011	1	0.918
Booklet group * T4	-0.071	0.086	-0.240	0.097	0.688	1	0.407
App group * T4	-0.209	0.134	-0.472	0.055	2.406	1	0.121
Triacylglycerols							
Booklet group	0.178	0.168	-0.151	0.508	1.125	1	0.289
App group	-0.023	0.169	-0.355	0.309	0.018	1	0.893
T4	-0.044	0.085	-0.211	0.122	0.271	1	0.603
Booklet group * T4	0.051	0.141	-0.226	0.327	0.128	1	0.720
App group * T4	0.128	0.167	-0.200	0.456	0.586	1	0.444
Fasting blood sugar							
Booklet group	-0.184	0.383	-0.935	0.568	0.230	1	0.632
App group	-0.429	0.336	-1.086	0.231	1.621	1	0.203
T4	-0.358	0.219	-0.788	0.073	2.649	1	0.104
Booklet group * T4	0.220	0.244	-0.259	0.699	0.808	1	0.369
App group * T4	0.218	0.243	-0.260	0.696	0.799	1	0.371
3-min step test							
Booklet group	-0.498	0.309	-1.105	0.109	2.581	1	0.108
App group	-0.256	0.331	-0.905	0.393	0.596	1	0.440
T4	0.078	0.149	-0.215	0.372	0.274	1	0.601
Booklet group * T4	0.026	0.219	-0.404	0.455	0.014	1	0.906
App group * T4	0.014	0.247	-0.472	0.500	0.003	1	0.955
Perceived stress scale							
Booklet group	-0.108	1.167	-2.396	2.179	0.009	1	0.926
App group	-2.192	1.309	-4.758	0.374	2.803	1	0.094

T2	-0.375	0.619	-1.589	0.839	0.367	1	0.545
T3	-0.327	0.746	-1.791	1.136	0.192	1	0.661
T4	0.783	1.457	-2.074	3.641	0.289	1	0.591
Booklet group * T2	-0.330	0.974	-2.240	1.580	0.115	1	0.735
Booklet group * T3	-0.087	1.110	-2.263	2.088	0.006	1	0.937
Booklet group * T4	-2.015	1.696	-5.340	1.310	1.411	1	0.235
App group * T2	1.058	0.903	-0.713	2.828	1.371	1	0.242
App group * T3	1.684	1.195	-0.658	4.027	1.985	1	0.159
App group * T4	-0.396	1.768	-3.862	3.070	0.050	1	0.823
Self-efficacy for exercise scale							
Booklet group	-0.520	0.412	-1.328	0.288	1.591	1	0.207
App group	0.198	0.436	-0.657	1.053	0.206	1	0.650
T2	0.203	0.338	-0.460	0.865	0.360	1	0.549
T3	-0.299	0.397	-1.078	0.481	0.565	1	0.452
T4	-0.336	0.431	-1.182	0.510	0.608	1	0.436
Booklet group * T2	0.341	0.489	-0.619	1.300	0.484	1	0.486
Booklet group * T3	0.595	0.532	-0.448	1.638	1.250	1	0.264
Booklet group * T4	0.607	0.537	-0.447	1.660	1.274	1	0.259
App group * T2	0.546	0.430	-0.297	1.389	1.609	1	0.205
App group * T3	1.043	0.499	0.064	2.022	4.360	1	0.037
App group * T4	1.170	0.542	0.106	2.235	4.648	1	0.031

GSLTPAQ = Godin-Shephard Leisure-Time Physical Activity Questionnaire, HDL = high density lipoprotein, LDL = low density lipoprotein.

Table S2. Results of generalized estimating equation analysis on outcome variables compared between app group and booklet group.

Variables	Beta	Standard Error	95% Confidence interval		Wald Chi-square	df	p-value
			lower	upper			
Body weight							
App group	-4.048	3.089	-10.103	2.006	1.717	1	0.190
T2	0.431	0.531	-0.611	1.474	0.658	1	0.417
T3	-0.298	0.516	-1.310	0.715	0.332	1	0.565
T4	-0.075	0.541	-1.135	0.985	0.019	1	0.890
App group * T2	-0.800	0.552	-1.882	0.283	2.095	1	0.148
App group * T3	-0.711	0.623	-1.933	0.511	1.301	1	0.254
App group * T4	-1.074	0.657	-2.362	0.214	2.671	1	0.102
GSLTPAQ							
App group	-0.891	3.261	-7.283	5.501	0.075	1	0.785
T2	-1.982	2.804	-7.479	3.515	0.499	1	0.480
T3	5.600	4.406	-3.037	14.236	1.615	1	0.204
T4	-3.311	2.456	-8.125	1.502	1.818	1	0.178
App group * T2	14.709	5.159	4.596	24.821	8.127	1	0.004
App group * T3	0.951	5.742	-10.304	12.205	0.027	1	0.869
App group * T4	14.709	5.159	4.596	24.821	8.127	1	0.001
Waist circumference							
App group	-3.477	2.097	-7.587	0.634	2.748	1	0.097
T4	-0.699	0.786	-2.241	0.844	0.788	1	0.375
App group * T4	-2.688	1.109	-4.862	-0.514	5.872	1	0.015
Systolic blood pressure							
App group	-0.024	3.321	-6.533	6.485	0.000	1	0.994
T4	-0.417	2.653	-5.618	4.783	0.025	1	0.875
App group * T4	-0.165	3.317	-6.667	6.336	0.002	1	0.960
Diastolic blood pressure							
App group	-2.440	1.964	-6.290	1.409	1.544	1	0.214
T4	-3.407	1.406	-6.164	-0.650	5.868	1	0.015
App group * T4	1.060	1.987	-2.835	4.955	0.285	1	0.594
Total cholesterol							
App group	-0.228	0.266	-0.751	0.294	0.735	1	0.391
T4	-0.140	0.078	-0.293	0.014	3.178	1	0.075
App group * T4	0.017	0.143	-0.265	0.299	0.014	1	0.905
HDL cholesterol							
App group	0.013	0.075	-0.135	0.162	0.031	1	0.860
T4	-0.054	0.028	-0.110	0.003	3.448	1	0.063
App group * T4	0.043	0.039	-0.035	0.121	1.179	1	0.278
LDL cholesterol							
App group	-0.151	0.226	-0.595	0.292	0.448	1	0.503

T4	-0.066	0.072	-0.209	0.076	0.842	1	0.359
App group * T4	-0.137	0.145	-0.422	0.148	0.887	1	0.346
Triacylglycerols							
App group	-0.201	0.178	-0.551	0.148	1.274	1	0.259
T4	0.006	0.112	-0.214	0.227	0.003	1	0.955
App group * T4	0.074	0.183	-0.284	0.433	0.165	1	0.684
Fasting blood sugar							
App group	-0.244	0.246	-0.727	0.238	0.983	1	0.321
T4	-0.132	0.107	-0.343	0.079	1.507	1	0.220
App group * T4	0.002	0.150	-0.292	0.296	0.000	1	0.988
3-min step test							
App group	0.242	0.301	-0.348	0.832	0.646	1	0.421
T4	0.104	0.159	-0.209	0.417	0.424	1	0.515
App group * T4	-0.012	0.253	-0.509	0.485	0.002	1	0.962
Perceived stress scale							
App group	-2.083	1.144	-4.327	0.160	3.313	1	0.069
T2	-0.712	0.753	-2.188	0.764	0.893	1	0.345
T3	-0.424	0.822	-2.036	1.189	0.265	1	0.607
T4	-1.241	0.869	-2.944	0.463	2.038	1	0.153
App group * T2	1.392	1.000	-0.568	3.351	1.937	1	0.164
App group * T3	1.775	1.243	-0.663	4.213	2.037	1	0.153
App group * T4	1.625	1.324	-0.972	4.222	1.505	1	0.220
Self-efficacy for exercise scale							
App group	0.718	0.395	-0.057	1.494	3.294	1	0.070
T2	0.543	0.353	-0.150	1.237	2.356	1	0.125
T3	0.296	0.353	-0.397	0.989	0.700	1	0.403
T4	0.271	0.320	-0.258	0.899	0.712	1	0.399
App group * T2	0.205	0.442	-0.663	1.073	0.215	1	0.643
App group * T3	0.448	0.465	-0.464	1.360	0.926	1	0.336
App group * T4	0.562	0.459	-0.338	1.463	1.498	1	0.221

GSLTPAQ = Godin-Shephard Leisure-Time Physical Activity Questionnaire, HDL = high density lipoprotein, LDL = low density lipoprotein.
